# miR-548aq-3p is a novel target of Far infrared radiation which predicts coronary artery disease endothelial colony forming cell responsiveness

**DOI:** 10.1038/s41598-020-63311-1

**Published:** 2020-04-22

**Authors:** Wei-Che Tsai, Wei-Hui Chiang, Chun-Hsien Wu, Yue-Cheng Li, Mel Campbell, Po-Hsun Huang, Ming-Wei Lin, Chi-Hung Lin, Shu-Meng Cheng, Pei-Ching Chang, Cheng-Chung Cheng

**Affiliations:** 1 Division of Cardiology, Department of Internal Medicine, Tri-Service General Hospital, National Defense Medical Center, Taipei, Taiwan; 2 Institute of Microbiology and Immunology, National Yang-Ming University, Taipei, Taiwan; 3 UC Davis Cancer Center, University of California, Davis, California USA; 4 Cardiovascular Research Center, National Yang-Ming University, Taipei, Taiwan; 5 Division of Cardiology, Department of Medicine, Taipei Veterans General Hospital and Institute of Clinical Medicine, Taipei, Taiwan; 6 Institute of Public Health, National Yang-Ming University, Taipei, 112 Taiwan; 7 Institute of Clinical Medicine, National Yang Ming University, Taipei, Taiwan; 8 Cancer Progression Research Center, National Yang-Ming University, Taipei, Taiwan

**Keywords:** Cell lineage, miRNAs

## Abstract

Non-invasive far infrared radiation (FIR) has been observed to improve the health of patients with coronary artery disease (CAD). Endothelial colony forming cells (ECFCs) contribute to vascular repair and CAD. The goal of this study was to uncover the role of FIR in ECFCs function and to reveal potential biomarkers for indication of FIR therapy in CAD patients. FIR significantly enhanced *in vitro* migration (transwell assay) and tube formation (tube length) capacities in a subpopulation of CAD ECFCs. Clinical parameters associated with the responsiveness of ECFCs to FIR include smoking and gender. ECFCs from CAD patients that smoke did not respond to FIR in most cases. In contrast, ECFCs from females showed a higher responsiveness to FIR than ECFCs from males. To decipher the molecular mechanisms by which FIR modulates ECFCs functions, regardless of sex, RNA sequencing analysis was performed in both genders of FIR-responsive and FIR-non/unresponsive ECFCs. Gene Ontology (GO) analysis of FIR up-regulated genes indicated that the pathways enriched in FIR-responsive ECFCs were involved in cell viability, angiogenesis and transcription. Small RNA sequencing illustrated 18 and 14 miRNAs that are up- and down-regulated, respectively, in FIR-responsive CAD ECFCs in both genders. Among the top 5 up- and down-regulated miRNAs, down-regulation of miR-548aq-3p in CAD ECFCs after FIR treatment was observed in FIR-responsive CAD ECFCs by RT-qPCR. Down-regulation of miR-548aq-3p was correlated with the tube formation activity of CAD ECFCs enhanced by FIR. After establishment of the down-regulation of miR-548aq-3p by FIR in CAD ECFCs, we demonstrated through overexpression and knockdown experiments that miR-548aq-3p contributes to the inhibition of the tube formation of ECFCs. This study suggests the down-regulation of miR-548aq-3p by FIR may contribute to the improvement of ECFCs function, and represents a novel biomarker for therapeutic usage of FIR in CAD patients.

## Introduction

Coronary artery disease (CAD) is the most prevalent type of cardiovascular disease (CVD) and the leading cause of death worldwide (http://www.wpro.who.int/china/ mediacentre/factsheets/cvd/en/). The impairment of endothelial integrity starts a cascade that leads to CAD^1^. Vessel maintenance and repair may not be solely dependent on proliferation of local endothelial cells (ECs), but also rely on bone marrow-derived endothelial progenitor cells (EPCs). The classification of EPC has been applied to multiple cell types, including endothelial colony forming cells (ECFCs)^2^. ECFCs exhibit the ability to form colonies after prolonged culture and proliferate after re-plating in an *in vitro* culture system. These cells are identified as positive for VE-cadherin, VEGFR2 (vascular endothelial growth factor receptor 2), VEGFR1, CD31, CD105, CD146, VWF (von Willebrand factor), CD34, and negative for CD14, CD45, CD115, CD133^3^. ECFCs have the potential to incorporate into the endothelial layer and differentiate into mature ECs, thereby contributing to repair of injured vascular endothelium as well as forming new blood vessels^4^. ECFCs can further enhance vasculogenic properties by releasing trophic factors^5^. With these capacities, ECFCs have been suggested to function in maintaining vascular homeostasis and preventing CAD. In line with this, increasing evidence shows that both the number and the angiogenic function of ECFCs are decreased significantly in CAD^6–10^. Thereby, ECFC transplantation has been reported experimentally to treat CAD^11–13^. However, the efficacy of ECFC transplantation is largely dependent on the functionality of the transplanted cells. ECFCs from CAD patients have impaired function and allogeneic ECFCs transplantation carries risks, including rejection. Therefore, physiotherapy with the potential to improve the function of the defective endogenous ECFCs could be an alternative therapy for CAD.

Far infrared radiation (FIR), a subdivision of infrared radiation (IR) in the wavelength of 3.0–1000 μm^14^, is a type of physiotherapy that can penetrate up to 4 cm (~1.5 inches) beneath the skin and generates both thermal and non-thermal effects on the human body^15^. FIR therapy has been shown to improve the health of patients with chronic diseases, in particular, vessel-related disorders^16^. The evidence shows that FIR treatment increased peripheral blood circulation^17^ and artery blood flow^18,19^ and thereby has been applied to improve access blood flow in hemodialysis patients^20–23^. FIR also has the potential for treatment of angiogenesis-related diseases include the improvement of ECs functions^24,25^, protection of ECs from high glucose-induced injury^26^, inhibition of EC inflammation^27^, and promotion of angiogenesis by ECs^28^. Importantly, FIR may also be beneficial for the function of ECFCs^29–32^. This includes studies investigating the role of FIR in ECFCs under stress conditions, especially high glucose/diabetes^30–32^. These studies showed that FIR significant reversed glucose-induced dysfunction of ECFCs. Mechanistically, FIR reversed the miRNA-134-NRIP1 axis, contributing to glucose-induced ECFCs dysfunction^32^. Moreover, our previous study also found that FIR can recover the function of CAD ECFCs by inducing miRNA-31 and miRNA-720^29^. These findings suggest the potential utility of FIR in the treatment of CAD.

In this study, we used *in vitro* cultivated ECFCs to examine the potential of using FIR as a therapeutic strategy for CAD patients. We demonstrate that FIR does not improve the function of ECFCs obtained from all CAD patients. Statistical analysis of FIR responsiveness with clinical parameters illustrated smoking as a negative correlate and female gender as more responsive to FIR treatment. RNA sequencing (RNA-seq) revealed ~700 and ~ 1300 genes differentially regulated by FIR in FIR-responsive and FIR-non/unresponsive ECFCs. Gene Ontology (GO) analysis showed that genes differentially regulated in FIR-responsive, but not FIR-non/unresponsive, ECFCs are enriched in pathways including cell viability, angiogenesis and transcription. Small RNA sequencing (smRNA-seq) followed by RT-qPCR identified miR-548aq-3p as a FIR suppressed molecule that correlated with the FIR responsiveness of CAD ECFCs. Through experiments of functional validation, we further demonstrated that overexpression of miR-548aq-3p inhibited the tube formation of healthy ECFCs. In contrast, knockdown of miR-548aq-3p enhanced the tube formation of CAD ECFCs, thus suggesting this miRNA may contribute to FIR-mediated improvement of ECFCs function.

## Materials and Methods

### ECFC isolation, cultivation and characterization

For the isolation of ECFCs, mononuclear cells (MNCs) were isolated from 16 ml of peripheral blood samples of individual healthy controls and CAD patients using Ficoll-Hypaque density gradient centrifugation (Lymphoprep, STEMCELL TECHNOLOGIES, Vancouver, British Columbia, CA). The isolated MNCs were resuspended in 4 ml of endothelial growth medium-2 (EGM2) with complete supplements (Lonza Ltd, Basel, CH) and seeded onto 2 wells of a 6-well plate coated with fibronectin (Merck Millipore, Burlington, MA, USA). After 2 weeks of cultivation at 37 °C and in 5% CO_2_, monolayer cells exhibited cobblestone-like morphology were obtained, reseeded and grown to confluence. ECFCs were characterized by flow cytometry (FACS) analysis (see below). All the ECFCs used in this study were cultured within six passages. The study of human specimens of healthy controls and CAD patients has been approved by the Institutional Review Board (IRB#: 1-107-05-155 and 2-108-05-097) of Tri-Service General Hospital. Written informed consent was obtained from all participants. The protocols of this study were consistent with the ethical guidelines of 1975 Helsinki Declaration.

### FACS analysis

ECFCs scraped by Corning cell lifter (Sigma, St. Louis, MO, USA) were suspended in PBS and 1 × 10^6^ cells were stained by specific antibodies at 4 °C for 20 minutes according to the manufacturer’s instructions. The antibodies used were FITC Mouse anti-Human CD34 (BD Pharmingen, Franklin Lakes, NJ, USA), FITC Mouse anti-Human CD31 (BD Pharmingen), FITC Mouse anti-Human CD45 (BD Pharmingen), FITC Mouse anti-Human CD144 (BD Pharmingen), PE Mouse anti-Human CD309 (BD Pharmingen), FITC Mouse IgG1, κ Isotype Control (BD Pharmingen), PE Mouse IgG1, κ Isotype Control (BD Pharmingen). To obtain single cell suspensions for FACS, cells were passed through a 40-μm cell strainer and transferred to a 5 ml polystyrene round-bottom Falcon tube (BD Pharmingen). ECFC surface antigens were detected by BD FACSCalibur (BD Biosciences, San Jose, CA) and the percentages of cell expressing the surface markers were calculated by FlowJo (BD Biosciences).

### Far infrared radiation (FIR) treatment

FIR treatment was done by exposure of human ECFCs to FIR radiation using a WSTM TY101 FIR emitter (WS Far Infrared Medical Technology Co., Ltd., Taipei, Taiwan). This device contains an electrified ceramic plate generating electromagnetic wavelengths of 3 to 25 μm (peak at 5 μm). The radiator was set at a height of 30 cm above the bottom of culture plates and the ECFCs were exposed to FIR radiation for 40 minutes. All assays were performed 24 hours after FIR treatment.

### RNA extraction and reverse transcription-quantitative polymerase chain reaction (RT-qPCR) assay

Total RNA, including small RNA, was isolated from ECFCs using miRNeasy Mini Kit (QIAGEN, Hilden, DE) following the manufacturer’s instructions. For miRNA detection, 100 ng of total RNA was reverse transcribed by miRCURY LNA miRNA PCR Starter kit (QIAGEN) and detected using miRCURY SYBR Green Master Mix (QIAGEN) in CFX96 connect real-time PCR detection system (Bio-Rad, Hercules, CA, USA). miRNA specific primers (Table S1) were purchased from miRCURY LNA miRNA PCR Starter kit (QIAGEN). The expression levels of miRNAs were normalized to UniSp6, a spike-in control small nuclear RNA.

### RNA-sequencing (RNA-seq) and gene ontology (GO) analysis

Total RNAs isolated from CAD ECFCs treated with and without FIR using miRNeasy Mini Kit (QIAGEN) were sequenced by 100-bp paired-end sequencing using an HiSeq 2500 (Illumina, San Diego, CA, USA) following the standard manufacturer’s instructions. Low quality sequences (<Q20) were removed and the adapters were trimmed by Bowtie. The remaining reads were aligned to Genome Reference Consortium Human Build 37 patch release 5 (GRCh37.p5) using CLC Genomics Workbench 10.0.1(QIAGEN). The expression values are presented as Reads Per Kilobase of transcript per Million mapped reads (RPKM). Ingenuity Pathway Analysis (IPA) software (http://www.ingenuity.com) was used to identify FIR altered gene expression in biological functions.

### Small RNA-sequencing (smRNA-seq) and data analysis

miRNAs isolated from CAD ECFCs before and after FIR treatment using miRNeasy Mini Kit (QIAGEN) were sequenced by HiSeq. 2500 (Illumina) following the standard manufacturer’s instructions. The CLC Genomics Workbench 11.0.1 (QIAGEN) was used to remove low quality sequences (<Q30) and to trim adapters from the smRNA-seq raw reads. The remaining reads were aligned to GRC37.p5 and annotation with miRBase v21 using CLC genomic workbench 11.0.1 (QIAGEN). The expression levels of miRNAs were calculated as reads per mapped reads (RPM).

### miRNA overexpression and knockdown

A genomic DNA fragment containing the precursor sequence of miR-548aq-3p was cloned into pLenti4-CMV/TO plasmid using miR-548aq-3p primer pairs: forward 5′-AAACGGTCCGCCCAGGTTCTTCACTGTTTC-3′ and reverse 5′-AAACGGACCGGCAGCCATTTTGGAAAGC-3′. Lentiviruses expressing miR-548aq-3p were produced by co-transfection of pLenti4-CMV/TO- miR-548aq-3p plasmid (0.4 μg) and packaging plasmids pCMV-VSV-G (0.1 μg) and pCMV-ΔR8.91 (0.3 μg) into 293 T cells using TransFectin lipid reagent (Bio-Rad). The lentiviruses were collected after 48 hours and used to transduce ECFCs. Infection was accomplished with the addition of polybrene at a final concentration of 8 μg/ml and spinfection at 1000 × *g* for 2 hours at room temperature (RT), followed by incubation at 37 °C for another 2 hours. 48 hours after transduction, ECFCs were used for further experiments.

To knockdown miR-548aq-3p in ECFCs, a commercial synthetic miRIDIAN microRNA Hairpin Inhibitor (hsa-miR-548aq-3p, IH-302531-01-0005) (Dharmacon, Lafayette, CO, USA) was added to the culture medium at a final concentration of 20 nM at 70~80% cell confluence using Lipofectamine RNAiMAX Transfection Reagent (Invitrogen, CA, USA). The expression level of miR-548aq-3p was measured by RT-qPCR after 48 hours.

### Cell migration and tube formation assays

For migration assays, EGM2 medium supplemented with 20%FBS (600 μl) was added to the lower chamber, while 5 × 10^4^ ECFCs in EGM2 medium (100 μl) were seeded on the upper chamber of Costar Transwell permeable supports (8 µm pore size, Corning, Corning, NY, USA). 4 hours after incubation at 37 °C, suspension cells were removed and the membranes were fixed with 4% paraformaldehyde for 20 minutes at RT. The migrated cells were stained with Hochest 33342 reagent (Sigma) for 5 minutes at RT and counted using fluorescence microscopy in ten random fields.

For *in vitro* tube formation assays, basement membrane extract (BME, Trevigen Inc., Gaithersburg, MD, USA) was coated in 96-well plates (50 μl/well) at 37 °C for 1 hour. Next, 5 × 10^3^ ECFCs in EGM2 medium supplemented with 20%FBS (100 μl) were placed onto the BME and incubated at 37 °C for 6 hours. 10 nM vinblastine (VB, Sigma) was added to the culture as negative control. The tube structures were visualized by inverted light microscope (100×) and ten representative fields were captured in each group. Total tube length was measured by ImageJ. Total number of tubes (>30 μm) and total number of branched tubes were counted.

### Statistical analysis

Categorical variables were analyzed using chi-square. Fisher’s exact test was performed when expected frequencies were <5. Difference between FIR treatments were analyzed using paired T-tests. Differences between FIR treatments in non-smoking male and female CAD ECFCs were analyzed using independent-samples t-test. Data is presented as mean + SD. All data was statistically analyzed using IBM SPSS Statistics version 24.

### Translational perspective

Coronary artery disease (CAD) is a leading cause of death worldwide. Identification of a non-invasive procedure that can restore the function of damaged blood vessel repairing cells is potentially important for the future treatment of CAD. However, in most cases these procedures face challenges related to individual differences in response to treatment. Precision medicine is an emerging approach that makes it possible to design highly effective personalized treatments. Far infrared radiation (FIR) is a type of physiotherapy that has been shown to improve the health of vessel-related disorders. Here, we identified miR-548aq-3p as a novel biomarker for therapeutic usage of FIR in CAD patients.

## Results

### Isolation and characterization of ECFCs from human peripheral blood

**Figure 1:**
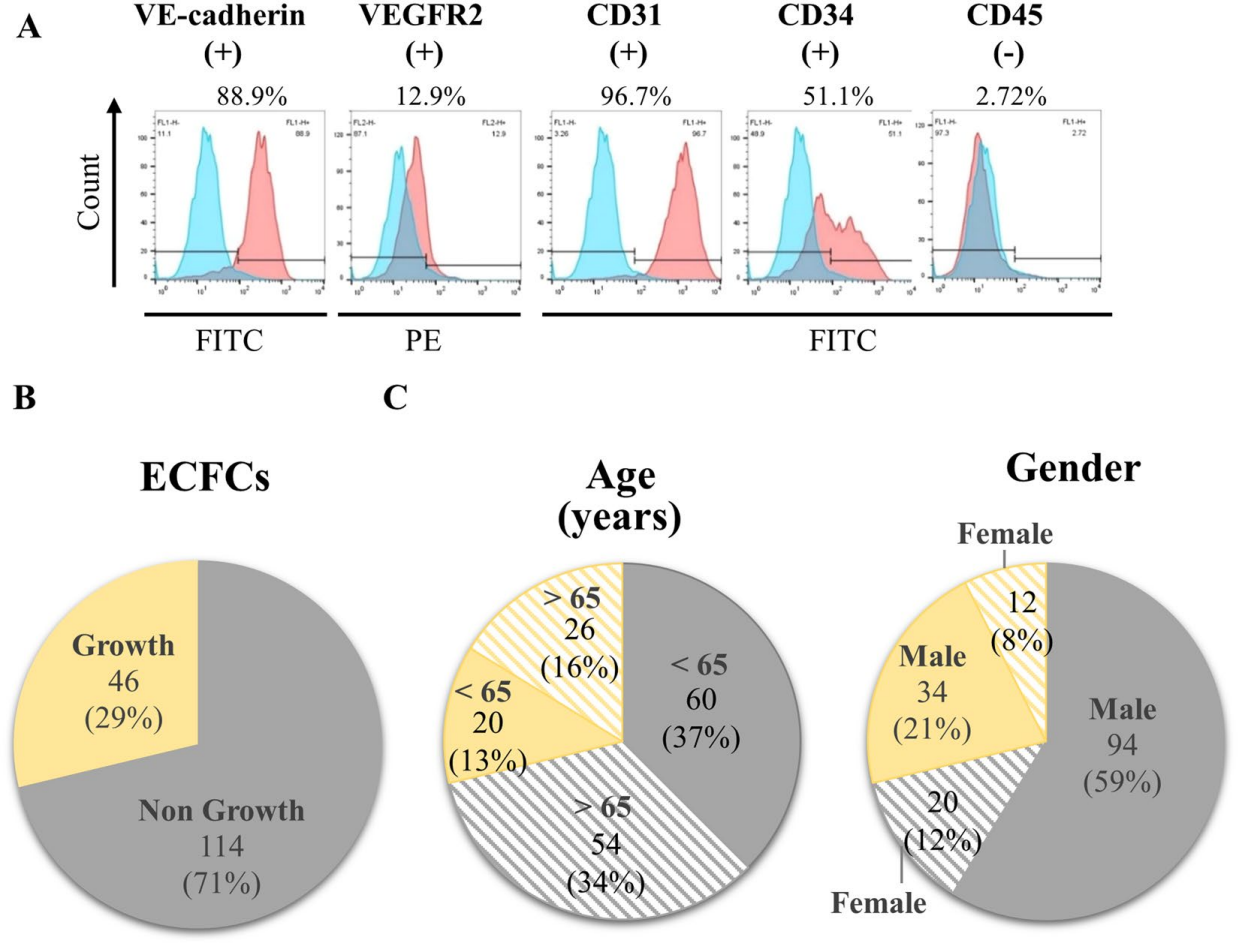
Isolation and characterization of ECFCs. (**A**) Representative flow cytometry immunophenotyping of ECFCs confirming the presence of VE-cadherin, VEGFR2, CD31 and CD34 and absence of CD45. Positive cells (%) for each marker are shown in the top of each panel. (**B**) Pie chart indicating the number and percentage (%) of ECFCs grown from 160 cultures of CAD patient mononuclear cells (MNCs). (**C**) Comparison of age (left panel) and gender (right panel) of CAD patients with the growth of ECFCs.

To isolate ECFCs, mononuclear cells (MNCs) obtained from the peripheral blood of healthy individuals and CAD patients were seeded on fibronectin-coated plates for 14 to 21 days. The colonies with cobblestone-like cells were subcultured twice and the amplified cells with a monolayer growth pattern were characterized by expression of endothelial markers VE-cadherin, VEGFR2, CD31 and CD34 but not hematopoietic marker CD45 (Fig. 1A). Following this procedure, 46 (29%) cultures of ECFCs were successfully isolated from peripheral blood samples of 160 CAD patients (Fig. 1B). The donor age and gender in related to the growth of ECFCs was statistically analyzed and showed no differences (Fig. 1C and Table 1).

**Table 1:** Comparison of age and gender with the growth of ECFCs.

Characteristic	All patients	Growth ECFCs		
		−	+	*p*-value
Age, years	160	114	46	0.295
<65	80	60	20	
>65	80	54	26	
Gender				0.221
Male	128	94	34	
Female	32	20	12	

### Effect of FIR on ECFCs function

**Figure 2:**
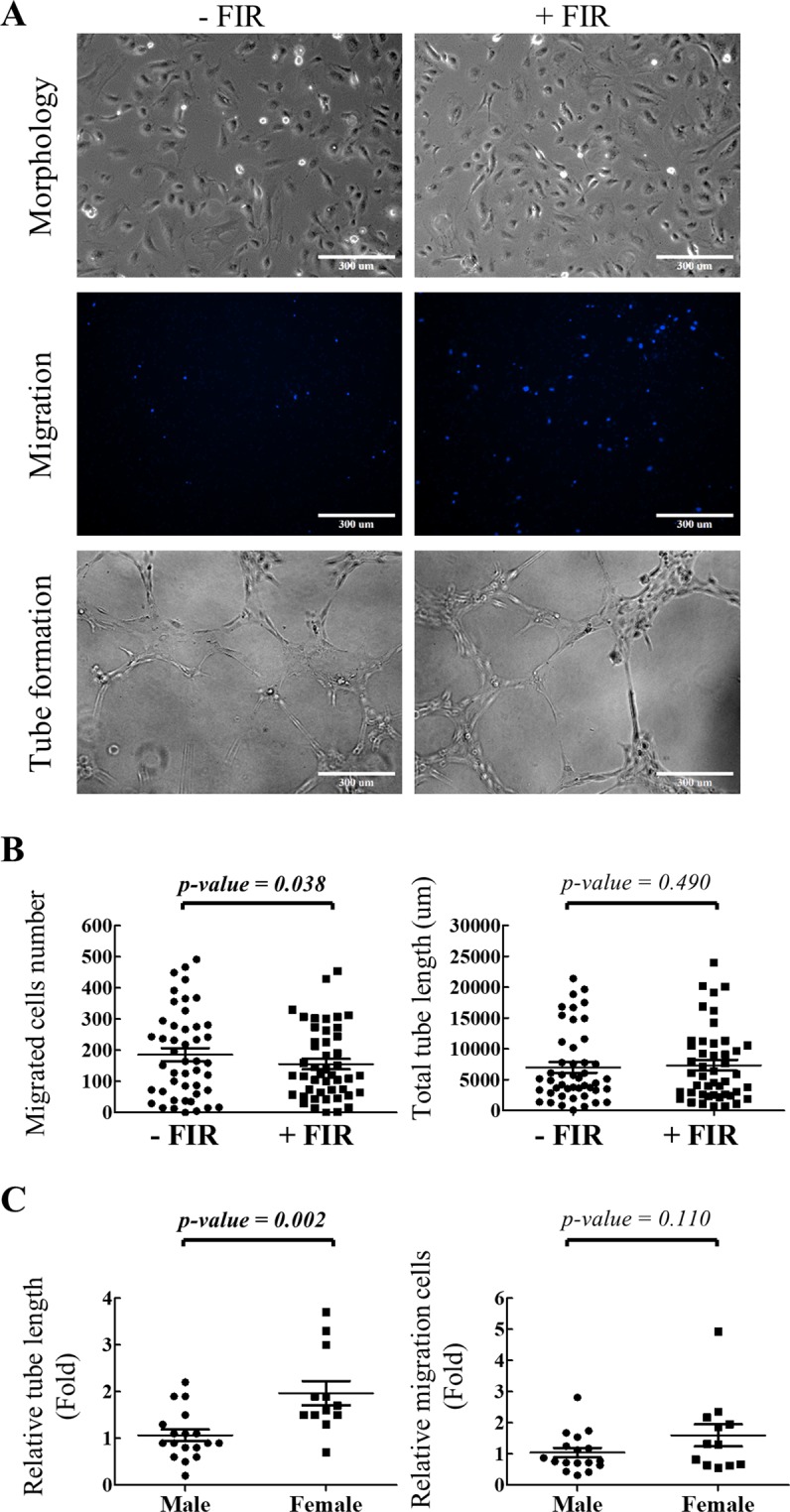
Effects of FIR treatment on CAD ECFC mobility and microvasculature formation *in vitro*. (**A**) Representative microscopic images showing the morphology (upper panel), migrated cells in the lower transwell chamber (middle panel) and tube formation on BME (lower panel) of CAD ECFCs before and after FIR treatment (original magnification 10×, scale bar 300 μm). (**B**) Statistical analysis of migrated cell number (left panel) and total tube length (right panel) of CAD ECFCs before and after FIR treatment. (n = 46) (**C**) Statistical analysis of relative tube length (left panel) and relative migrated cell number (right panel) of non-smoking male (n = 18) and female (n = 12) CAD ECFCs before and after FIR treatment. (*Student’s-t* test).

To study if repeated FIR treatment can ameliorate the function of CAD ECFCs, ECFC cultures derived from CAD patients (n = 8) were exposed to FIR treatment with an intensity of 10 mW/cm^2^ for 40 minutes once daily for 1 or 3 days (Fig. S1A). The cell surface markers of ECFCs were measured by flow cytometry and showed no significant difference (Fig. S1B). The migration and tube formation ability of ECFCs were then analyzed and no significant differences between the 2 treatment groups were observed (Fig. S1C). Therefore, a single FIR treatment for 40 minutes was used to evaluate FIR effects on the migration and tube formation function of the 46 cultivated CAD ECFCs. 24 hours after treatment, cell migration and tube formation was measured. Among the 46 CAD ECFC cultures, only 10 and 17 exhibited increased cell migration and tube formation, respectively (Fig. 2A). However, when the results of all 46 CAD ECFCs were plotted, cell migration seems to be inhibited by FIR treatment (Fig. 2B, left panel) and tube formation showed no statistical difference (Fig. 2B, right panel).

### Female gender and smoking are the strongest predictors of CAD ECFCs responsiveness to FIR treatment

The result above prompted us to hypothesize that response to FIR treatment may vary between ECFC cultures based on unknown individual differences that exist among the CAD ECFC donors. We therefore further evaluated the impact of clinical risk factors on the responsiveness of ECFCs to FIR. Clinical characteristics of the 46 CAD patients are summarized in Table 2. There were no statistically significant differences identified in term of age, cardiovascular risk factors, chronic kidney disease (CKD) and diabetes mellitus (DM) between the FIR-treated and -untreated groups (Table 2). In CAD ECFCs treated with FIR, the change in cell migration and tube formation were significantly higher in female donors versus male (Table 2). In addition, the change in CAD ECFCs migration ability was significantly higher in non-smoking donors than smoking (Table 2).

**Table 2:** Comparison of clinical characteristics of CAD ECFCs with FIR responsive.

Characteristic	All patients	Migration			Tube formation		
		−	+	*p*-value	−	+	*p*-value
**Age, years**	46	36	10	***0.726***	29	17	0.809
**<65**	20	15	5		13	7	
**>65**	26	−1	5		−6	10	
**Gender**				***0.012***			***<0.001***
Male	34	30	4		27	7	
Female	12	6	6		2	10	
**Involved diseased coronary arteries**				0.493			0.999
1-Vessel	8	5	3		5	3	
2-Vessel	11	9	2		7	4	
3-Vessel	27	22	5		17	10	
**Coronary artery disease history**				0.475			0.760
−	23	17	6		15	8	
+	23	19	4		14	9	
**Dyslipidemia**				***1.000***			0.708
−	20	16	4		12	8	
+	26	20	6		17	9	
**Acute coronary syndrome**				−			−
−	46	36	10		29	17	
+	0	0	0		0	0	
**Stroke / Transient ischemic attack**				***1.000***			***1.000***
−	44	34	10		28	16	
+	2	2	0		1	1	
**Family history**				***1.000***			***1.000***
−	20	33	9		26	16	
+	26	3	1		3	1	
**Hypertension**				***1.000***			***1.000***
−	7	6	1		4	3	
+	39	30	9		25	14	
**Diabetes mellitus (DM)**				***1.000***			0.708
−	26	20	6		17	9	
+	20	16	4		12	8	
**Smoking**				***0.009***			0.062
−	30	20	10		16	14	
+	16	16	0		13	3	
**Chronic kidney disease (CKD)**				***1.000***			***0.443***
−	38	30	8		25	13	
+	8	6	2		4	4	

*Black: Chi-Square Tests, Bold & Italic: Fisher’ Exact test*.

It should be noted that all CAD patients that smoke are male. We therefore re-evaluated the FIR responsiveness in non-smoking CAD ECFCs. Statistical analysis showed that the change in CAD ECFCs tube formation was consistently significantly higher in female than male (Fig. 2C, left panel), but this was not statistically significant for cell migration (Fig. 2C, right panel). This result indicated that smoking might reduce the potential for FIR induced migration of CAD ECFCs. In addition, female gender is an independent factor for FIR responsiveness in the tube formation ability of ECFCs. However, we cannot exclude the influence of male gender since 7 of the 34 CAD ECFCs from male (~20%) also showed responsiveness to FIR treatment. These results together support our hypothesis that the variability of CAD ECFC response to FIR treatment reflects individual donor differences.

### Transcriptome profiling to identify pathways involved in FIR treatment

To reveal the signaling pathways behind the FIR response, RNA-seq was performed in one male (CAD178) and one female (CAD162) CAD ECFCs that showed an increase in tube formation after FIR treatment and one male (CAD222) and one female (CAD239) FIR-non/unresponsive CAD ECFCs (Fig. S2). The expression of endothelial markers VE-cadherin, VEGFR2, VEGFR1, CD31, CD34, CD105, CD146, VWF and hematopoietic markers CD14, CD45, CD115, CD133 were first extracted from the RNA-seq data (Table S2). The high expression of endothelial markers and low levels of hematopoietic markers confirmed the characteristics of ECFCs (Fig. S1D).

**Figure 3:**
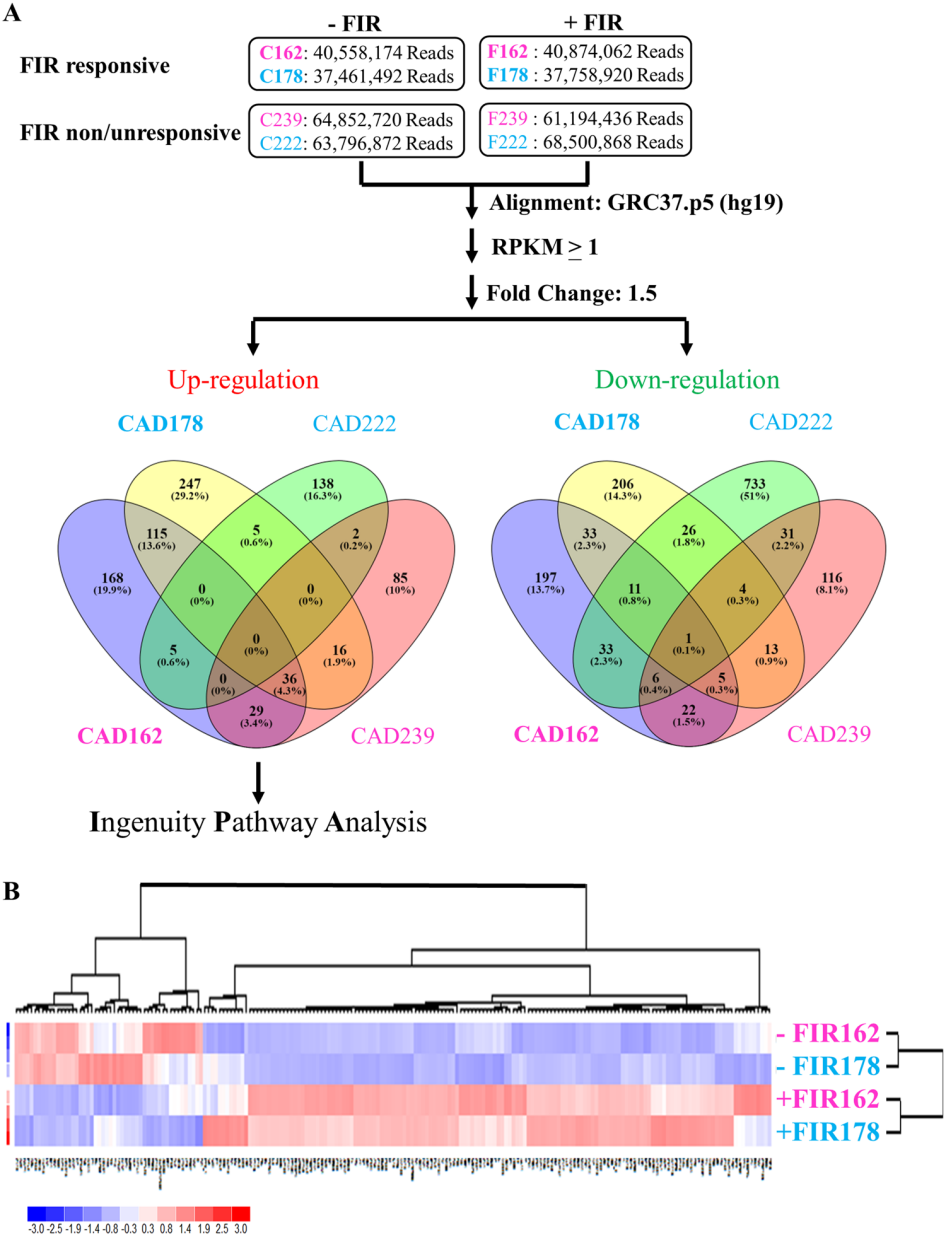
FIR treatment changes gene expression in FIR-responsive and FIR-non/unresponsive male and female CAD ECFCs. (**A**) Summary of RNA-seq data. FIR-responsive and FIR-non/unresponsive CAD ECFCs were treated with FIR. 24 hours after FIR treatment, total RNA was extracted and used for RNA-seq on Illumina HiSeq2500. Reads Per Kilobase per Million mapped reads (RPKM) higher than 1 in at least one sample were considered as expressed genes and used for fold-change calculations. Venn diagram showing the mRNAs that are up- or down-regulated more than 1.5 fold by FIR treatment. Blue (Male) and Pink (Female). Boldface (FIR-responsive) and Light face (FIR-non/unresponsive). (**B**) Heatmap of hierarchical cluster analysis of mRNAs simultaneously up- (151) and down-(50) regulated in FIR-responsive male and female CAD ECFCs after FIR treatment.

As shown in Fig. 3A, we identified 419 and 353 mRNAs (RPKM > 1) that were up-regulated in FIR-responsive male and female CAD ECFCs, respectively. In addition, 299 and 308 mRNAs were down-regulated in FIR-responsive male and female CAD ECFCs (Fig. 3A, right panel). For FIR-non/unresponsive CAD ECFCs, 150 and 168 mRNAs were up-regulated (Fig. 3A, left panel) and 845 and 198 mRNAs were down-regulated (Fig. 3A, right panel) in male and female, respectively. When the differentially expressed genes in FIR-responsive male and female CAD ECFCs were compared, there are 151 and 50 mRNAs that were up- and down-regulated, respectively, in both genders. Unsupervised hierarchical clustering analysis of expression of the 201 simultaneous up- and down-regulated mRNAs showed that FIR-treated and un-treated samples were grouped in separate clusters (Fig. 3B), demonstrating that FIR may regulate a group of genes regardless of gender.

To explore the potential mechanisms of response to the FIR treatment, genes that were up-regulated by FIR in FIR-responsive male (419 mRNAs) and female (353 mRNAs) CAD ECFCs (Fig. 3A, left panel) were subjected to Gene Ontology (GO) analysis using Ingenuity Pathway Analysis (IPA) software. We used an activation *z*-score to identify the downstream signaling pathways modulated by FIR and considered a *z*-score of >2 as significant activation of the functional activity identified. Consistent with previous reports showing that FIR enhances cell survival and migration of ECs from human^26,28^ and bone-marrow-derived stem cells (BMSCs) from rat^33^, biological processes including cell viability and angiogenesis were identified (Table 3). However, when the genes that were up-regulated by FIR in FIR-non/unresponsive male (150 mRNAs) and female (168 mRNAs) CAD ECFCs (Fig. 3A) were subjected to GO analysis using IPA, only cell viability were identified in the female CAD ECFCs (Table 3, left panel). These GO analyze suggest that FIR may improve the function of CAD ECFCs through modulation of cell angiogenesis.

**Table 3:** Gene Ontology (GO) analysis of FIR in modulating the function of CAD ECFCs.

Diseases or Functions Annotation	FIR			
	Responsive		Non/Unresponsive	
	162	178	239	222
**Cell Death and Survival**				
Cell viability	*4.79*	*5.74*	*3.44*	—
**Cardiovascular System Development and Function**				
Angiogenesis	*2.03*	*2.60*	—	—
**Gene Expression**				
Expression of RNA	*3.57*	*2.27*	—	—
Transcription	*4.14*	*2.53*	—	—
Transcription of DNA	*3.31*	*2.00*	—	—
Transcription of RNA	*3.56*	*2.12*	—	—

### smRNA-seq analysis identifies miR-548aq-3p which is decreased in FIR-treated female CAD ECFCs

**Figure 4:**
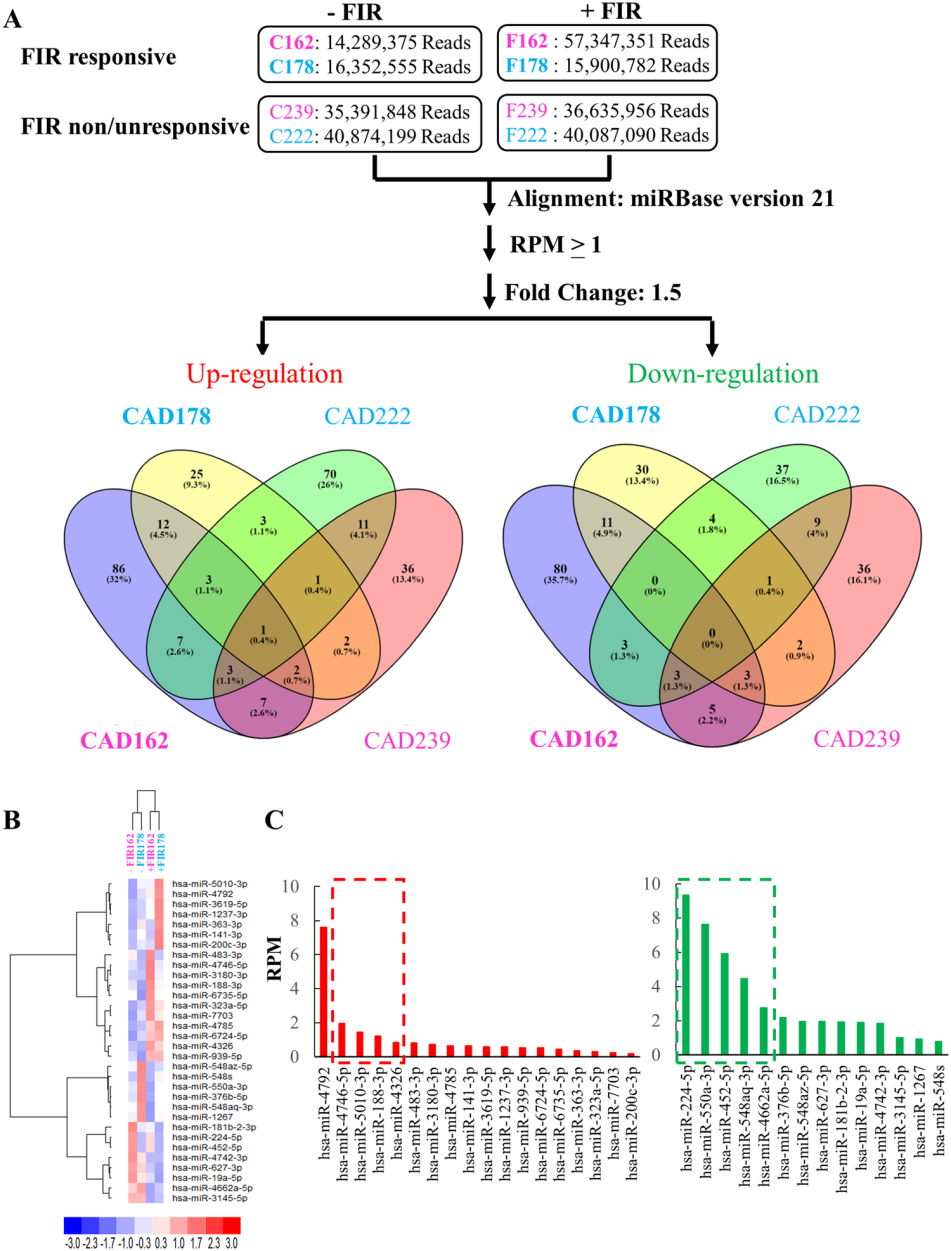
smRNA-seq data analysis. (**A**) Flowchart of the miRNA analysis pipeline. Total RNA prepared from Fig. 3A was subjected to smRNA-seq. Read per millions of mapped reads (RPM) higher than 1 in at least one sample were considered as expressed miRNAs and used for fold-change calculations. Venn diagram showing the miRNAs that are up- or down-regulated more than 1.5-fold by FIR treatment. Colors as described in Fig. 3A. (**B**) Heatmap of hierarchical cluster analysis of miRNAs simultaneously up- (18) and down-(14) regulated in FIR-responsive male and female CAD ECFCs after FIR treatment. (**C**) Histogram demonstrating the expression level of the FIR up- (left panel) and down- (right panel) regulated miRNAs in (**B**).

Following our previous studies demonstrating that miRNAs may participate in FIR-mediated beneficial effects on ECFCs^29,32^, we next analyzed the differences of miRNAs expression patterns between FIR-responsive and –non/unresponsive CAD ECFCs by smRNA-seq using small RNAs isolated from the same male and female CAD ECFCs used in the RNA-seq analysis. We identified 49 and 121 miRNAs (RPM > 1) that were up-regulated in FIR-responsive male and female CAD ECFCs, respectively (Fig. 4A, left panel). In addition, 51 and 111 miRNAs were down-regulated in FIR-responsive male and female CAD ECFCs (Fig. 4A, right panel). For FIR-non/unresponsive CAD ECFCs, 99 and 63 miRNAs were up-regulated (Fig. 4A, left panel) and 57 and 59 miRNAs were down-regulated (Fig. 4A, right panel) in male and female, respectively. It should be noticed that little miRNAs were commonly up- or down-regulated by FIR-responsive and –non/unresponsive CAD ECFCs (Fig. 4A). In addition, we noticed that more miRNAs (>2-fold) were differentially regulated by FIR in FIR-responsive female ECFCs when compared with male ECFCs. This phenomenon was not observed in mRNAs, suggesting the higher response of female CAD ECFCs to FIR may, in part, due to the differential expression of miRNAs.

**Figure 5:**
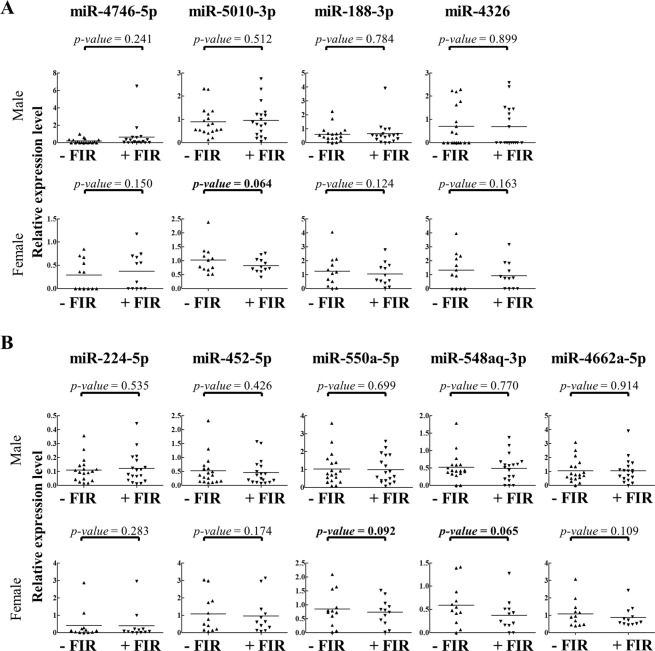
Reverse transcription-quantitative polymerase chain reaction (RT-qPCR) reveals a slight down-regulation of miR-548aq-3p and miR-550a-5p in FIR-treated CAD ECFCs. (**A**) The top 4 FIR-induced miRNAs were validated in CAD ECFCs after FIR treatment. (**B**) The top 5 FIR-suppressed miRNAs were validated as described in (**A**). (n = 30; Paired *Student’s-t* test). Bold, 0.05 < *p*-value < 0.1.

**Figure 6:**
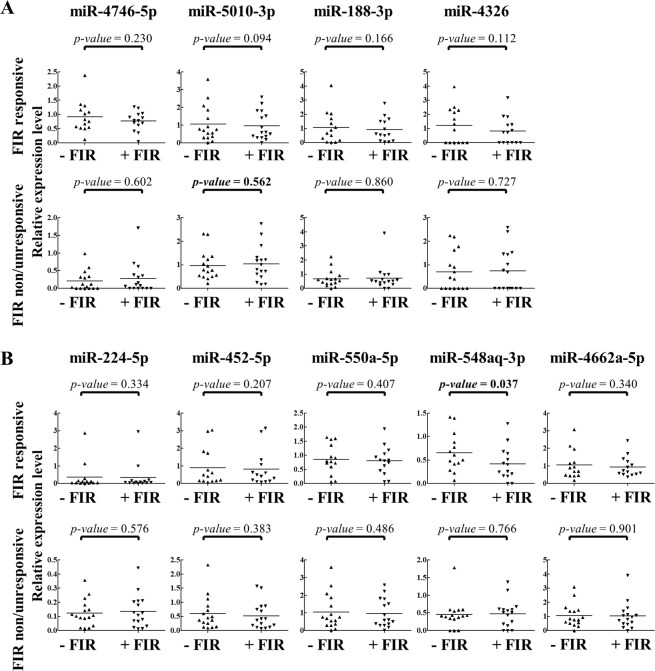
RT-qPCR reveals a significant down-regulation of miR-548aq-3p in FIR-responsive CAD ECFCs after FIR treatment. (**A**) The top 4 FIR-induced miRNAs were validated in 16 FIR-non/unresponsive and 14 FIR-responsive CAD ECFCs after FIR treatment. (**B**) The top 5 FIR-suppressed miRNAs were validated as described in (**A**). (n = 30; Paired *Student’s-t* test). Bold, *p*-value < 0.05.

To identify miRNAs that were up- or down-regulated by FIR in both FIR-responsive male and female CAD ECFCs, the differentially expressed miRNAs were compared and 18 and 14 simultaneously up- and down-regulated miRNAs, respectively, were revealed (Fig. 4A). Unsupervised hierarchical clustering analysis of expression of these 32 miRNAs showed that FIR treated and un-treated samples were grouped in separate clusters (Fig. 4B), demonstrating that FIR may also regulate a number of miRNAs regardless of gender. The top 5 most highly expressed up- and down-regulated miRNAs was chosen for further validation (Fig. 4C). However, since miR-4792, the most highly expressed up-regulated miRNA, was recently annotated to 28S rRNA sequences (http://www.mirbase.org/cgi-bin/mirna_entry.pl?acc=MI0017439) and removed from the miRBase v22 database, miRNA RT-qPCR assays were performed in 4 up-regulated and 5 down-regulated miRNAs using small RNAs isolated from the 18 non-smoking male and 12 female CAD ECFCs (Fig. 3C) treated with or without FIR. None of the 4 up-regulated miRNAs from smRNA-seq data showed significant up-regulation in FIR-treated male and female CAD ECFCs (Fig. 5A). For the 5 down-regulated miRNAs, miR-550a-5p and miR-548aq-3p were decreased in female CAD ECFCs after FIR treatment with borderline statistical significance (Fig. 5B). Fisher’s exact test showed that the down-regulation of miR-548aq-3p in CAD ECFCs after FIR treatment was significantly correlated with tube formation activity (Table 4). When the non-smoking CAD ECFCs were grouped by tube formation responsiveness to FIR treatment, miR-548aq-3p was statistically significant down-regulated after FIR-treatment (Fig. 6). Moreover, when clinical characteristics were taken into consideration, down-regulation of miR-548aq-3p by FIR was observed in all chronic kidney disease (CKD) with statistical significance (*p* < 0.01) (Table 5) and CAD ECFCs from 3 of the 4 CKD patients showed increased tube formation activity after FIR treatment. These data together suggest that miR-548aq-3p may be a potential biomarker for evaluating the efficacy of FIR therapy in CAD patients with CKD.

**Table 4:** The correlation between miR-548aq-3p and miR-550a-5p expression and ECFCs functions.

Characteristic	Non smoking	miR-548aq-3p			miR-550a-5p		
		−/↑	↓	*p*-value	−/↑	↓	*p*-value
**Migration**	30	21	9	0.389	29	1	1.000
**−**	21	16	5		20	1	
+	9	5	4		9	0	
**Tube formation**				**0.046**			1.000
**−**	16	14	2		15	1	
+	14	7	7		14	0	

*Fisher’ Exact test*.

*Bold: p*-value < 0.05.

^**−**/**↑**^The miRNA was not down-regulated after FIR treatment.

^**↓**^The miRNA was down-regulated after FIR treatment.

**Table 5:** The correlation between miR-548aq-3p and miR-550a-5p expression and clinical factors.

Characteristic	Non smoking	miR-548aq-3p			miR-550a-5p		
		−/↑	↓	*p*-value	−/↑	↓	*p*-value
**Age, years**				1.000			1.000
**<65**	12	8	4		12	0	
**>65**	18	13	5		17	1	
**Gender**				0.102			1.000
male	18	15	3		17	1	
female	12	6	6		12	0	
**Involved diseased coronary arteries**				0.766			0.673
1-Vessel	4	3	1		4	0	
2-Vessel	9	7	2		9	0	
3-Vessel	17	11	6		16	1	
**Coronary artery disease history**				1.000			0.467
**−**	16	11	5		16	0	
+	14	10	4		13	1	
**Dyslipidemia**				0.687			0.367
**−**	11	7	4		10	1	
+	19	14	5		19	0	
**Acute coronary syndrome**				**−**			**−**
**−**	30	21	9		29	1	
+	0	0	0		0	0	
**Stroke / Transient ischemic attack**				0.083			0.067
**−**	28	21	7		28	0	
+	2	0	2		1	1	
**Family history**				1.000			1.000
**−**	27	19	8		26	1	
+	3	2	1		3	0	
**Hypertension**				1.000			1.000
**−**	4	3	1		4	0	
+	26	18	8		25	1	
**Diabetes mellitus (DM)**				0.236			1.000
**−**	16	13	3		15	1	
+	14	8	6		14	0	
**Smoking**				**−**			**−**
**−**	30	21	9		29	1	
+	0	0	0		0	0	
**Chronic kidney disease (CKD)**				**0.005**			0.133
**−**	26	21	5		26	0	
+	4	0	4		3	1	

*Fisher’ Exact test*.

*Bold: p*-value < 0.05.

^**−**/**↑**^The miRNA was not down-regulated after FIR treatment.

^**↓**^The miRNA was down-regulated after FIR treatment.

### Identification of miR-548aq-3p as a potential angiogenic miRNA

**Figure 7:**
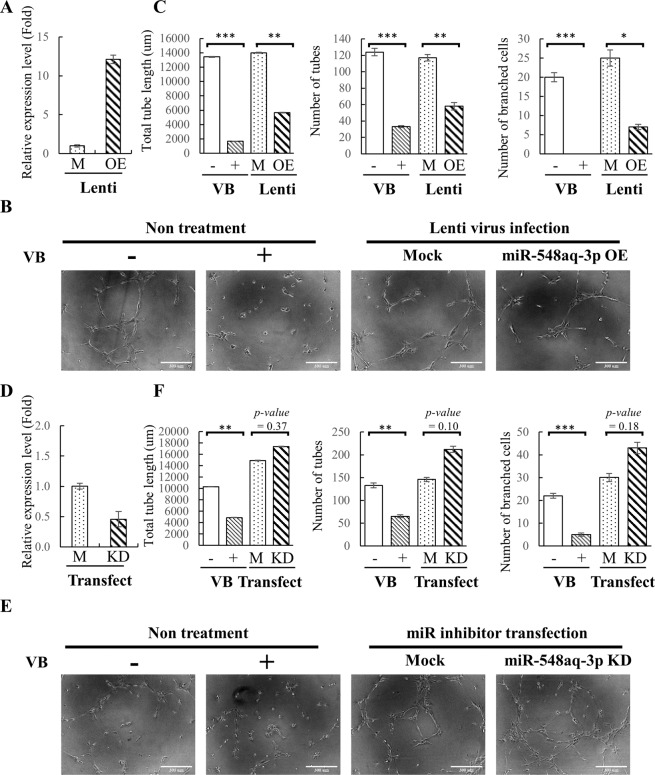
Functional characteristics of miR-548aq-3p as anti-angiogenic miRNAs. (**A**) Healthy ECFCs were transduced with mock lentivirus (M) or lentivirus overexpressing miR-548aq-3p (OE). 48 hours after transduction, total RNA was extracted and the expression levels of miR-548aq-3p were quantified by RT-qPCR. (**B**) Representative images from the tube formation assays of non-transduced controls treated with or without 10 nM vinblastine (VB), mock infected (M) and miR-548aq-3p overexpressed (OE) healthy ECFCs (original magnification 10×, scale bar 300 μm). (**C**) Quantitative data of total tube length (left panel), number of tubes (>30 μm) (middle panel) and number of branched cells (right panel) in (**B**). (**D**) CAD ECFCs were transfected with mock (M) or miR-548aq-3p inhibitor (KD) using Lipofectamine RNAiMAX. 48 hours after transfection, total RNA was extracted and the expression levels of miR-548aq-3p were quantified by RT-qPCR. (**E**) Representative images from the tube formation assay of CAD ECFCs treated with or without 10 nM vinblastine (VB), mock transfected (M) and miR-548aq-3p knockdown (KD) (original magnification 10×, scale bar 300 μm). (**F**) Quantitative data of total tube length (left panel), number of tubes (>30 μm) (middle panel) and number of branched cells (right panel) in (**E**).

We then investigated the effect of miR-548aq-3p on ECFCs activities. To this end, a genomic DNA fragment containing the precursor sequence of miR-548aq-3p was cloned into a lentiviral-based expression vector, pLenti4-CMV/TO-Flag. Overexpression of miR-548aq-3p in healthy ECFCs following transduction of a lentiviral vector expressing miR-548aq-3p (Fig. 7A) was found to significantly inhibit tube formation activities (Fig. 7B,C). Consistent with this result, knockdown of miR-548aq-3p (Fig. 7D) by miRIDIAN microRNA Hairpin Inhibitor slightly increased tube formation of CAD ECFCs (Fig. 7E,F).

**Figure 8:**
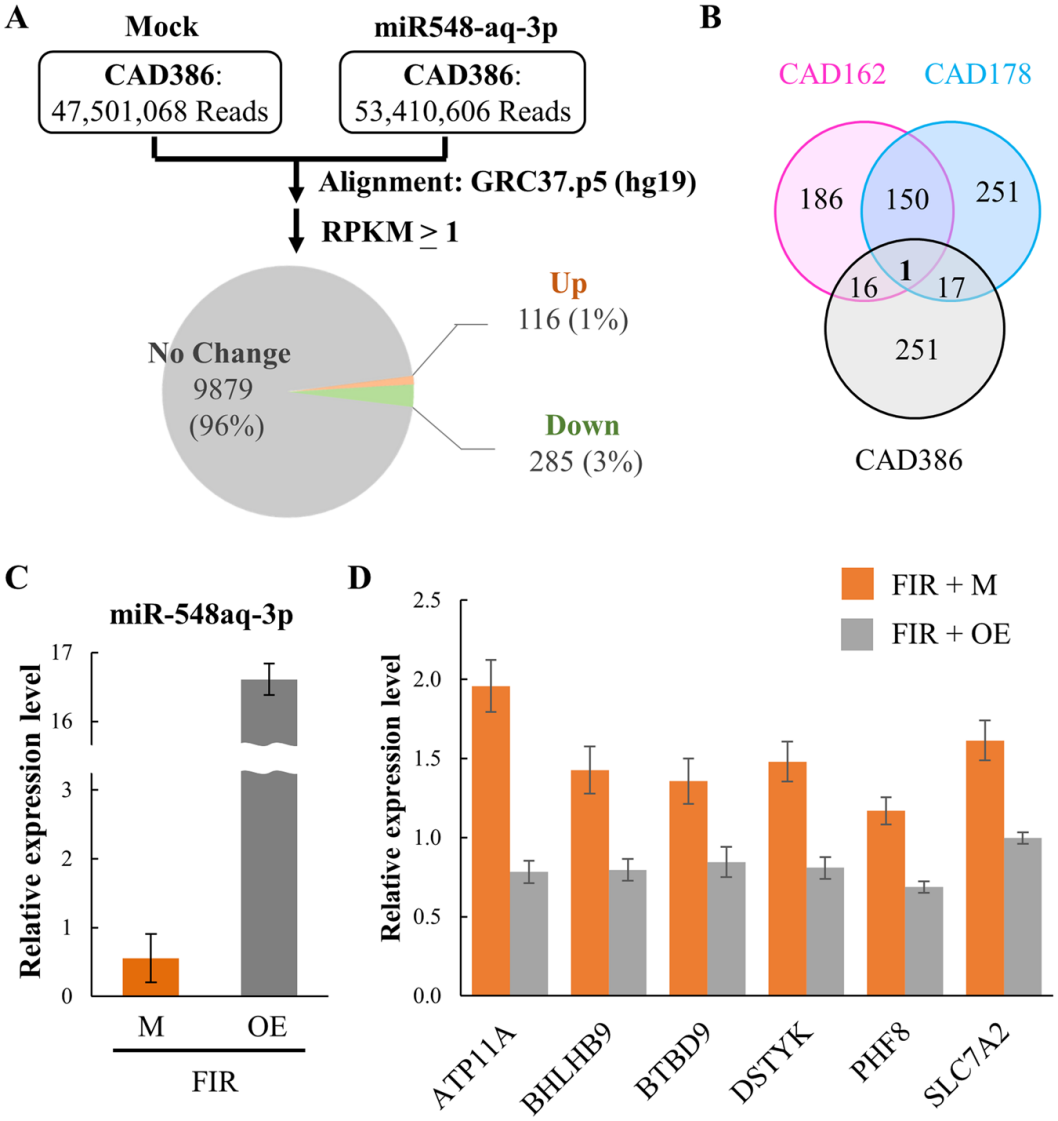
Identification of potential target genes of miR-548aq-3p. (**A**) Summary of RNA-seq data. Total RNA extracted from control and miR-548aq-3p overexpressed CAD ECFCs was used for RNA-seq on Illumina HiSeq. 2500. RPKM higher than 1 in at least one sample are considered as expressed genes and used for fold-change calculations. Pie chart showing the mRNAs that are up- or down-regulated more than 1.5 fold by miR-548aq-3p overexpression. (**B**) Venn diagram showing the mRNAs that are up-regulated by FIR treatment (pink and blue) and down-regulated by miR-548aq-3p overexpression (Gray). (**C**) CAD ECFCs were transduced with mock lentivirus (M) or lentivirus overexpressing miR-548aq-3p (OE). 48 hours after transfection, total RNA was extracted and the expression levels of miR-548aq-3p were quantified by RT-qPCR. (**D**) RT-qPCR analysis using RNA prepared from (**C**) demonstrating increased expression of ATP11A, BHLHB9, BTBD9, DSTYK, PHF8, and SLC7A2 after FIR treatment which was decreased by miR-548aq-3p overexpression (OE).

To identify possible targets of miR-548aq-3p, we performed another RNA-seq in control and miR-548aq-3p overexpressed CAD ECFCs. The results showed that only 3% of genes were down-regulated by miR-548aq-3p overexpression (Fig. 8A). By overlapping the genes that were up-regulated by FIR treatment (Fig. 3) and the genes that were down-regulated by miR-548aq-3p overexpression, 34 potential miR-548aq-3p targeted genes were identified (Fig. 8B and Table 6). TargetScan 7.1 bioinformatic algorithm was used to predict potentially miR-548aq-3p target sites. Among the 34 genes, 23 were identified to be potential targeted by at least one miR-548 family member, containing identical seeding sequence to miR-548aq-3p (Fig. S3). To further validate the *in silico* findings, the expression levels of the 23 potential miR-548 target genes were analyzed in control and miR-548aq-3p overexpressed CAD ECFCs using RT-qPCR (Fig. 8C). The expression of ATP11A, BHLHB9, BTBD9, DSTYK, PHF8, and SLC7A2 were increased after FIR treatment and repressed by miR-548aq-3p overexpression (Fig. 8D). This suggests that these genes are potential targets of miR-548aq-3p.

**Table 6:** Summary of RNA-seq and RT-qPCR results of potential miRNA-548 targeted genes.

Gene symbol	TargetScan7.1	CAD386			CAD162			CAD178			RT-qPCR	
		miR5480E		Down	FIR		UP	FIR		UP	FIR	
		Mock	OE	(1.5×)	−	+	(1.5×)	−	+	(1.5×)	Mock	OE
ATF3	**−**	1.17	0.22	0.18	3.67	3.66		1.80	3.18	+	**−**	**−**
**ATP11A**	hsa-miR-548ae-3p	2.03	1.18	0.58	3.19	5.24	+	5.83	8.37		1.96	0.78
**BHLHB9**	hsa-miR-548ae-3p	1.17	0.01	0.01	0.80	0.69		0.59	1.31	+	1.43	0.80
**BTBD9**	hsa-miR-548ae-3p	1.16	0.71	0.61	1.58	1.70		1.25	2.03	+	1.36	0.85
CCDC103	hsa-miR-548ae-3p	2.23	1.27	0.57	0.46	1.02	+	1.67	1.52		1.02	0.84
CYLD	hsa-miR-548ae-3p	1.41	0.19	0.14	2.86	4.39	+	4.30	5.72		1.11	0.95
DNAJA4	**−**	3.45	2.03	0.59	6.16	6.45		2.03	3.60	+	**−**	**−**
DPY19L3	hsa-miR-548ae-3p	3.09	1.61	0.52	3.14	5.12	+	1.63	1.49		1.04	0.92
**DSTYK**	hsa-miR-548ae-3p	1.07	0.54	0.51	2.06	3.35	+	2.44	3.29		1.48	0.81
FIGNL1	hsa-miR-548ae-3p	2.17	0.01	0.00	2.63	5.12	+	4.16	2.46		1.28	1.00
FKTN	hsa-miR-548ae-3p	1.50	0.79	0.53	1.57	3.24	+	2.13	2.58		1.06	0.80
GSTT2B	**−**	4.30	1.79	0.42	0.43	1.12	+	4.66	3.21		**−**	**−**
HIST1H2BC	**−**	1.14	0.65	0.57	3.06	5.31	+	3.05	4.07		**−**	**−**
HIST2H4B	**−**	1.60	0.31	0.19	1.03	0.88		0.48	1.09	+	**−**	**−**
HYAL3	**−**	2.11	1.04	0.49	1.35	2.22	+	1.73	1.67		**−**	**−**
MECOM	hsa-miR-548ae-3p	####	6.76	0.66	9.52	7.83		5.48	8.79	+	0.83	0.77
MUTYH	**−**	3.58	1.90	0.53	0.99	1.57	+	1.00	0.01		**−**	**−**
OASL	**−**	4.87	3.05	0.63	2.59	2.65		1.12	2.33	+	**−**	**−**
PEX26	hsa-miR-548ae-3p	3.80	1.68	0.44	3.20	6.09	+	4.23	5.42		0.97	0.62
PHF7	hsa-miR-548ae-3p	1.26	0.57	0.45	1.13	0.38		0.74	1.23	+	0.81	0.83
**PHF8**	hsa-miR-548ae-3p	1.58	0.74	0.47	2.52	2.60		1.44	3.00	+	1.17	0.69
RAD17	**−**	5.08	1.97	0.39	6.66	5.10		4.09	6.62	+	**−**	**−**
RBBP8	hsa-miR-548ah-3p	2.20	0.79	0.36	1.94	2.24		2.06	3.10	+	0.80	0.74
**SLC7A2**	hsa-miR-548ae-3p	1.83	0.79	0.43	6.27	7.51		3.18	5.53	+	1.61	1.00
SMC2	hsa-miR-548ae-3p	1.13	0.74	0.65	1.62	2.57	+	1.45	4.59	+	0.99	0.79
SPAST	hsa-miR-548ae-3p	2.92	1.88	0.64	2.43	5.28	+	3.42	4.95		0.83	0.80
STIM2	hsa-miR-548ae-3p	1.49	0.01	0.01	2.46	2.61		1.89	3.49	+	1.11	1.47
TCP11L1	**−**	2.41	1.31	0.54	2.98	4.48	+	3.96	4.89		**−**	**−**
TLK1	hsa-miR-548ae-3p	2.16	0.34	0.16	4.29	0.02		4.07	6.85	+	1.22	0.88
WDHD1	hsa-miR-548ae-3p	1.07	0.04	0.04	1.67	1.96		1.52	2.77	+	0.84	1.00
XIAP	hsa-miR-548j-3p	2.25	1.27	0.56	5.21	6.09		2.88	6.00	+	1.13	1.03
XRCC3	**−**	2.11	0.82	0.39	0.97	1.83	+	2.23	0.85		**−**	**−**
ZBTB16	hsa-miR-548ae-3p	1.09	0.65	0.60	1.09	2.51	+	1.40	1.69		1.15	1.50

*Bold: genes listed in* Fig. 8D.

## Discussion

By using 46 CAD ECFCs isolated from CAD patients, the effect of FIR on the functionality of ECFCs was evaluated. Statistical analysis indicated that FIR showed a higher improvement in tube formation ability of female ECFCs when compared with male ECFCs. Bioinformatics analysis of transcriptome data showed that FIR may modulate the angiogenesis function of CAD ECFCs. smRNA-seq revealed down-regulation of miR-548aq-3p may represent a unique mechanism to improve the tube formation ability of ECFCs following FIR treatment.

Though previous reports including ours showed the beneficial effect of FIR on circulating ECFCs^29–32^, an extensive screening for the application of FIR in CAD ECFCs has never been performed. Surprisingly, FIR improves cell migration (10/46; 22%) and tube formation (17/46; 37%) in only a small population of CAD ECFCs (Table 2). Thus it is not unexpected that the statistical analysis showed no significant improvement of CAD ECFCs migration and tube formation ability by FIR treatment (Fig. 2B). This suggests the effect of FIR therapy on circulating ECFCs may vary dependent upon individual donor differences. In line with this, we evaluated the ECFCs responsiveness to FIR with clinical factors and identified gender and smoking as two discriminant factors for FIR treatment. Smoking significantly reduced the incidence of cell migration (0% compared to 33.3% of non-smoker) and tube formation (18.8% compared to 46.7% of non-smoker) ability of ECFCs enhanced by FIR treatment (Table 2). This observation indicates that FIR intervention may not be an effective option for CAD patients that smoke. However, whether this effect is permanent or reversible after cessation of smoking is an interesting topic for further study.

We noticed that all of the 16 smoking subjects in this study are male. This prompted us to speculate that the lower FIR responsiveness we identified in male ECFCs may come from the smoking population. Therefore, the FIR responsiveness in the non-smoking CAD ECFCs was re-evaluated by gender (Fig. 2C). Importantly, a significant higher improvement in tube formation ability of ECFCs by FIR treatment persists in female (age 68.4 + 9.5) when compared with male (age 62.8 + 9.3) (Fig. 2C). It should be noted that all female CAD subjects in this study are postmenopausal. It has been long-known that, due to the loss of the protective effects by hormone, CAD risk increased in postmenopausal women^34–36^. Interestingly, one recent report suggested that reduction of circulating nitric oxide (NO) may account for the decrease in the number and dysfunction of ECFCs in postmenopausal hypercholesterolemic women^37^. Since FIR was known to up-regulate endothelial NO production^24,25^, our findings may link FIR recovery of CAD ECFCs activities in postmenopausal women through increasing NO production. This is also an interesting topic for future study.

miRNAs are the well-known small RNA species of approximately 20 nucleotides (nts) that participate in post-transcriptional regulation of gene expression. Previously, we deciphered the miRNA transcriptome (miRNome) patterns in ECFCs using smRNA-seq and illustrated several aspects of miRNAs in regulating the biological function of ECFCs. These included: **(1)** increased microtubule formation ability of peripheral blood-derived ECFCs by angiogenic miR-31 that is highly expressed in cord blood-derived ECFCs^38^; **(2)** reduction in the microtubule formation ability of ECFCs by anti-angiogenic miR-221, miR-222, miR-146a-5p and miR-146b-5 that are up-regulated in CAD^39,40^; **(3)** direct targeting of endothelial mitogen vascular endothelial growth factor (VEGF) by anti-angiogenic miR-361-5p which diminishes the function of ECFCs^41^; and **(4)** targeting VEGF receptor 2 (VEGFR2) by anti-angiogenic miR-410-3p, miR-497-5p, and miR-2355-5p which reduces ECFCs functions^42^. These studies highlight the function of miRNAs and their role in the pathogenesis of CAD. To identify the FIR-modulated miRNAs in CAD ECFCs, irrespective of sex, we performed smRNA-seq in cultures of one male and one female FIR-responsive and FIR-non/unresponsive ECFCs. The top 5 miRNAs that were up- (except for miR-4792) and down-regulated in FIR-responsive ECFCs after FIR treatment was chosen for RT-qPCR analysis in the 30 non-smoking CAD ECFC cDNAs. Among them, only two miRNAs (miR-550a-5p and miR-548aq-3p) showed a borderline statistically significant reduction in expression levels (Fig. 5B). This suggests FIR modulation of miRNAs may also vary among individuals. In line with this, when the down-regulation of miR-548aq-3p and miR-550a-5p were correlated with the functional data of ECFCs, miR-548aq-3p down-regulation was significantly related to tube formation (Fig. 6 and Table 4).

miR-548aq-3p is a novel miRNA with no biological function as yet defined. To elucidate if miR-548aq-3p is involved in the function of ECFCs, we performed miR-548aq-3p overexpression and knockdown experiments in healthy and CAD ECFCs, respectively. Overexpressing miR-548aq-3p inhibited and knockdown miR-548aq-3p enhanced the tube formation ability of ECFCs (Fig. 7). It is therefore possible that the decreased level of miR-548aq-3p upon FIR treatment may be responsible for the enhanced functions observed in ECFCs. However, the role of miR-548aq-3p in the molecular mechanisms by which FIR functions in ECFC biology remains to be further investigated.

Identification of biomolecules that confer CAD ECFCs responsiveness to FIR may help to improve the clinical application of FIR therapy or may be used as biomarkers for precision medicine. Here, we found that FIR-mediated down-regulation of miR-548aq-3p in CAD ECFCs may represent a unique mechanism to augment the function of ECFCs by FIR therapy for CAD patients. Deciphering FIR-affected miRNAs provides us with new knowledge regarding angiogenesis.

## Supplementary information

Supplementary Information.
