# Supplementary material for: miR-548aq-3p is a novel target of Far infrared radiation which predicts coronary artery disease endothelial colony forming cell responsiveness
Source: Sci Rep. 2020 Apr 22;10:6805. doi: 10.1038/s41598-020-63311-1 (PMC7176637; doi:10.1038/s41598-020-63311-1)
Supplement: Supplementary file 1 — Supplementary Information. [file 41598_2020_63311_MOESM1_ESM.pdf]

miR-548aq-3p is a novel target of Far infrared radiation which predicts coronary artery disease endothelial colony forming cell responsiveness

Wei-Che Tsai[a,b*], Wei-Hui Chiang[b*], Chun-Hsien Wu[a,b], Yue-Cheng Li[b], Mel Campbell[c], Po-Hsun Huang[d,e], Ming-Wei Lin[f], Chi-Hung Lin[b,g], Shu-Meng Cheng[a], Pei-Ching Chang[b,h‡], Cheng-Chung Cheng[a‡]

[a]Division of Cardiology, Department of Internal Medicine, Tri-Service General Hospital, National Defense Medical Center, Taipei, Taiwan
[b]Institute of Microbiology and Immunology, National Yang-Ming University, Taipei, Taiwan
[c]UC Davis Cancer Center, University of California, Davis, California, USA
[d]Cardiovascular Research Center, National Yang-Ming University, Taipei, Taiwan
[e]Division of Cardiology, Department of Medicine, Taipei Veterans General Hospital and Institute of Clinical Medicine, Taipei, Taiwan.
[f]Institute of Public Health, National Yang-Ming University, Taipei 112, Taiwan
[g]Institute of Clinical Medicine, National Yang Ming University, Taipei, Taiwan
[h]Cancer Progression Research Center, National Yang-Ming University, Taipei, Taiwan
[‡]Corresponding author

[*]First author
[‡]Corresponding author: Pei-Ching Chang and Cheng-Chung Cheng

Address correspondence to: Pei-Ching Chang
Institute of Microbiology and Immunology, National Yang-Ming University, Taipei 11221, Taiwan. Phone: +886-2-2826-7111; E-mail: pcchang@ym.edu.tw

Address correspondence to: Cheng-Chung Cheng
Division of Cardiology, Department of Internal Medicine, Tri-Service General Hospital, National Defense Medical Center, Taipei, Taiwan. Phone: +886-2-87923311 ext12837; Fax: +886-2-66012656 Email: chengcc@ndmctsgh.edu.tw

Short Title: miR-548aq-3p in CAD ECFCs responsiveness to FIR

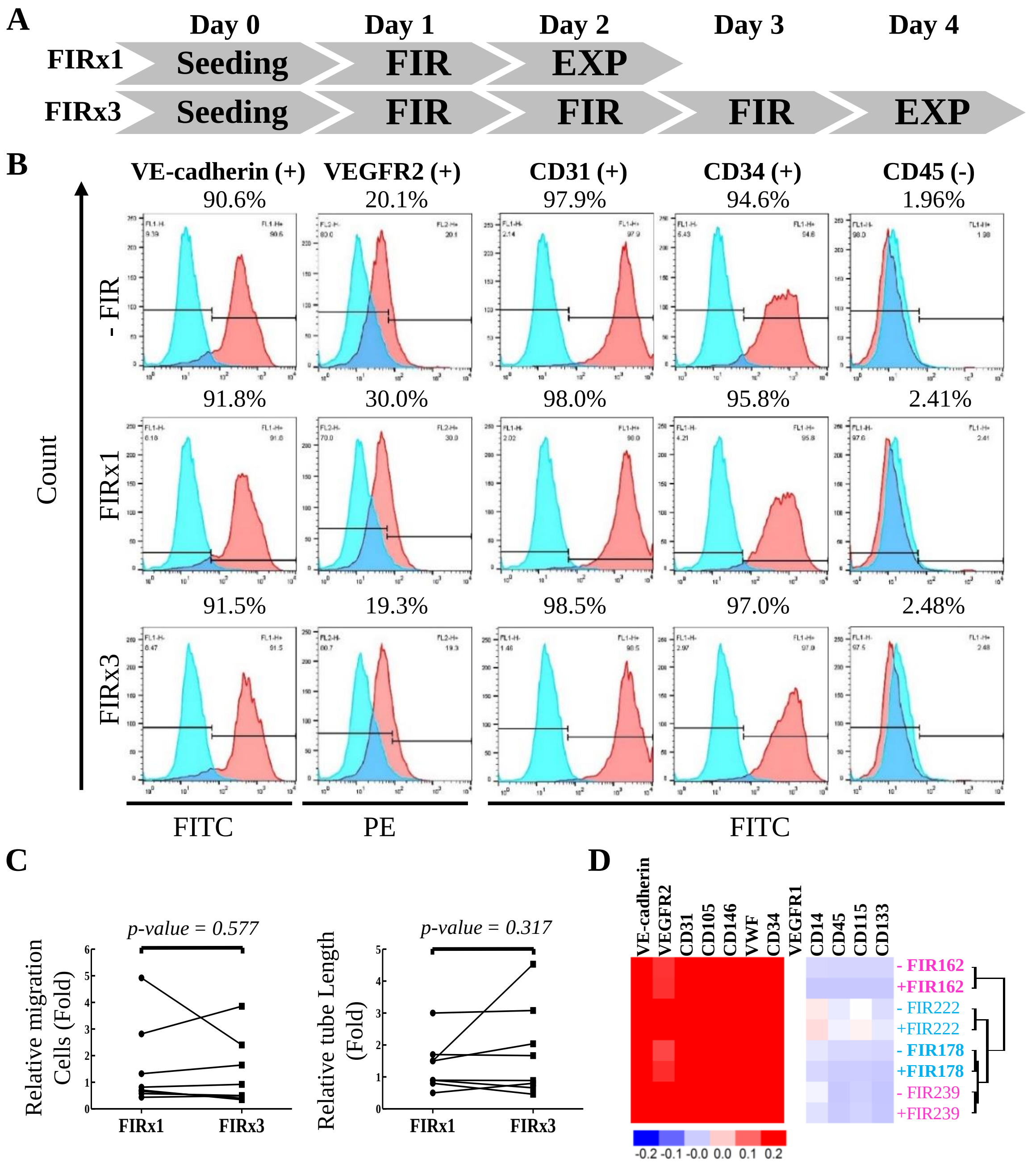

Figure S1. Repeated FIR treatment does not further improve the function of CAD ECFCs. (A) Schematic of CAD ECFCs treated with one (FIRx1) or three (FIRx3) cycles of FIR. (B) Immunophenotyping of ECFCs as described in Figure 1A. Representative flow cytometry plot of CAD ECFCs before and after one or three FIR treatments. (C) Statistical analysis of migrated cell number (left panel) and tube length (right panel) of CAD ECFCs after one or three FIR treatments relative to control un-treated ECFCs. (n=8, Paired *Student's-t* test). (D) Heatmap showed the expression of endothelial markers VE-cadherin, VEGFR2, VEGFR1, CD31, CD34, CD105, CD146, VWF and hematopoietic markers in FIR-responsive and FIR-unresponsive male and female CAD ECFCs before and after FIR treatment.

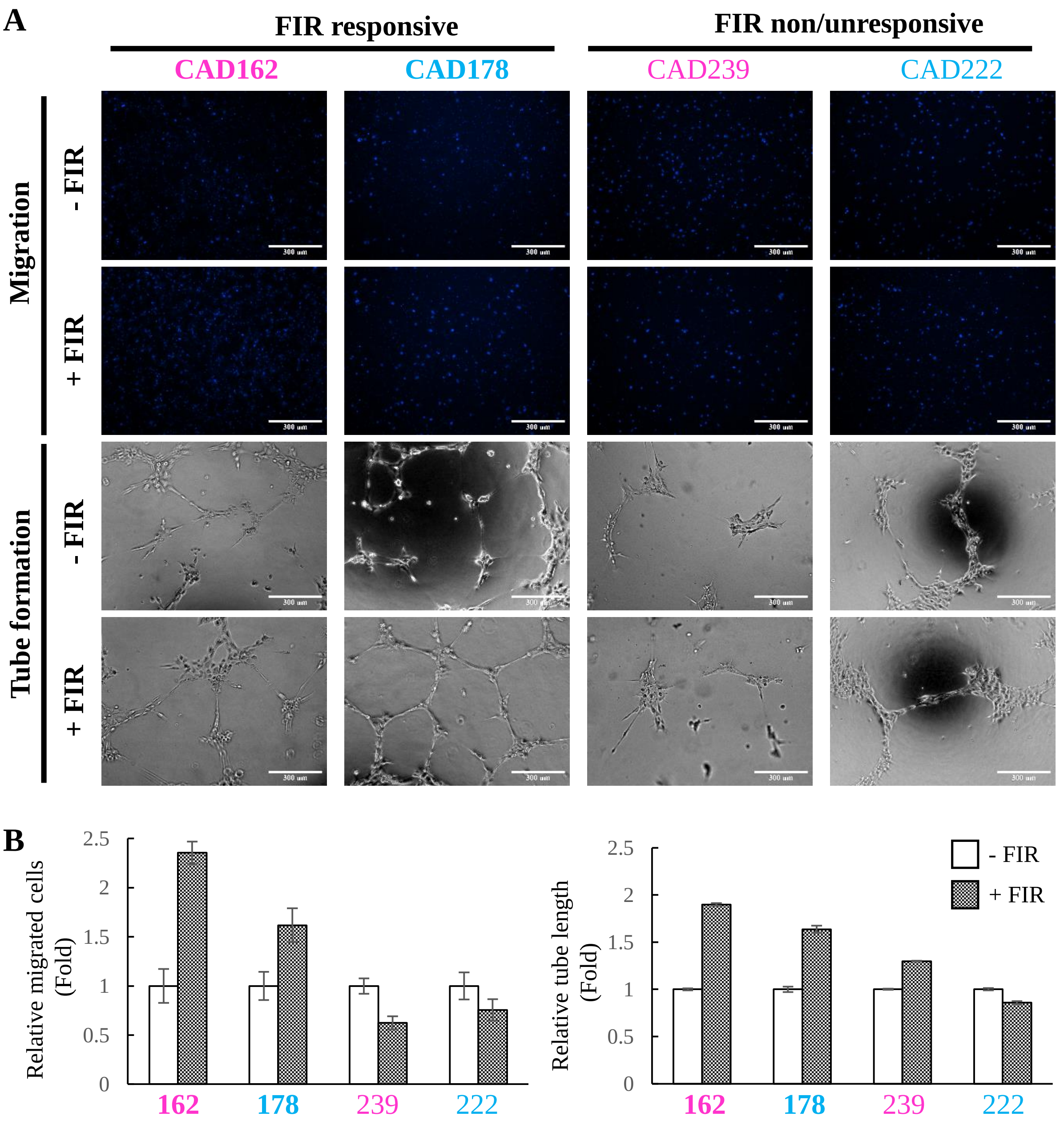

Figure S2. Mobility and microvasculature formation of 2 FIR-responsive and 2 FIR-unresponsive CAD ECFCs used for RNA-seq analysis. (A) Representative microscopic images showing the migrated cells in the lower transwell chamber (upper panel) and tube formation on BME (lower panel) of CAD ECFCs before and after FIR treatment (original magnification 10x, scale bar 400 μm). (B) Quantitative data of relative tube length (upper panel) and migrated cell number (lower panel).

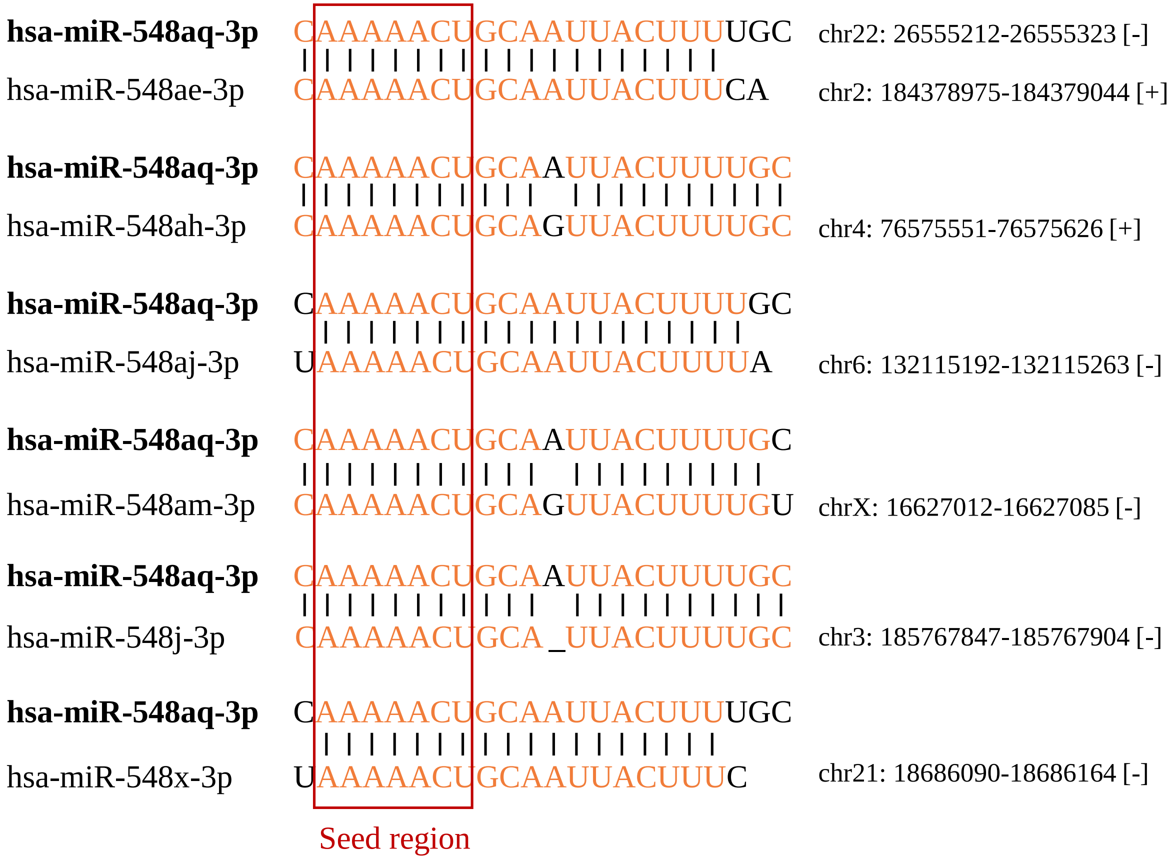

Figure S3. Sequence alignment of miR-548aq-3p with the other miR-548 family members showing the identical seed region.

Table S1 miRCURY LNA miRNA primer list

Brand	Name	Cat. No.
QIAGEN	hsa-miR-188-3p	YP02119296
	hsa-miR-224-5p	YP00204641
	hsa-miR-4326	YP02103754
	hsa-miR-452-5p	YP00204301
	hsa-miR-4662a-5p	YP02115325
	hsa-miR-4746-5p	YP02113549
	hsa-miR-5010-3p	YP02109984
	hsa-miR-550a-3p	YP00204795
	hsa-miR-548aq-3p	YP02100851

Table S2 The expression levels of endothelial and hematopoietic markers in CAD ECFCs

Marker	CAD ECFCs								Colony forming unit – Hill	Circulating angiogenic cell	Endothelial colony forming cell
	(-) FIR				(+) FIR						
	162	178	239	222	162	178	239	222			
VE-cadherin	296.95	297.09	301.37	241.52	281.73	302.24	295.37	266.36	+/-	+/-	++
VEGFR2	49.04	48.98	95.74	47.73	48.13	53.36	79.05	54.57	+	+	++
CD31	433.31	270.61	251.55	307.60	395.38	248.35	316.90	266.23	+	+	+
CD105	352.42	346.29	266.37	230.65	350.25	345.55	279.64	242.86	+	+	+
CD146	710.68	959.94	672.02	628.69	637.99	888.10	705.94	623.38	+/-	+/-	+
VWF	196.64	188.17	410.96	136.27	174.39	184.52	369.12	163.30	+/-	+/-	+
CD34	576.37	171.69	248.35	67.30	551.77	181.63	282.41	65.52	+/-	+/-	+/-
VEGFR1	8.53	9.08	5.97	8.72	10.17	10.93	4.13	8.27	+	+	+
CD14	0.35	3.63	9.76	7.00	0.04	2.92	10.27	3.73	+	+	-
CD45	0.01	0.78	2.35	0.62	0.07	0.78	1.72	0.58	+/-	+/-	-
CD115	0.01	0.80	5.74	1.75	0.01	0.94	6.42	1.89	+	+	-
CD133	0.01	0.13	0.20	0.07	0.01	0.03	0.11	0.01	+	+	-

Table S3 Primer sequences for RT-qPCR used in this study

Gene	Forward primer (5'-3')	Reverse primer (5'-3')
ERN1	GCGAACAGAATACACCATCAC	GCATCACCTTCCTCAGACC
FRS2	TGTCCCAGATAACCATCGG	GGCAAAGATTCCTTGTCCA
ITGAV	ACAATGAAGCCTTAGCAAGAC	GCACACTGAAACGAAGACC
KLF7	GTGAGCCAGACAGACTGAC	ATTCAAGGCATGTCTGCTGC
MGAT5	TGTGGCAGATATCATTAACGG	TCTGTACGAATTTCCGCCA
NF1	TCCTGCTCTGTATCCAATGCT	CACACGAACATACCTGACCA
PRKAA1	AGCCGAGAAGCAGAAACAC	TTCATGTTTGCCAACCTTCAC
PRKACB	GGACCTTCTACGGAACCTG	GGAGCTTCAACCTTCCTCTG
RGS4	AGTCATGAATGTGGGCTGG	GGAATCCTTCTCCATCAGGT
ROCK2	GACACAAACAGGAACTTACGG	TCTCAGCCAACTTATTCACAG
TBX20	ATTGAGAGGGAAAGTGTGGA	GGAATGGGTGTTGCTATGG
ATP11A	TCTACAACATCAGCTTCACCT	AGTTTCCAAATATCTGCCCGT
BHLHB9	ATGTTAAGGAGCTCAAGAAGC	TCTCTTTCCAAACATACAGCC
BTBD9	TGCCAGTGTGATTGAAGGA	AGTAGTAACCGTATTGACCCA
CCDC103	CATCACATCTGAAGCCACTG	GAAGTCCAAATCCCACATCTG
CYLD	CAAGTCCACCTTCATCCGA	ATTCTGACCACCATCCCGA
DPY19L3	GGACCAGAGCGGTTTATCAG	GATCTCTTCACAGAAGCGAGG
DSTYK	CTGTTGAATCTGCTGTTGGG	AACGTCCACTTCCTGTAAGAG
FIGNL1	GCAGATGATAGAGCCAGGA	GATGCTGCTATCTCAGTACCA
FKTN	TTTGACAGGCCAGAGTTACAG	ACCATCCTAGACAAGTTCCAC
MECOM	CCCAGTGAGGTATAAAGAGGA	CTGCGATTTGGACTTTCTGTG
PEX26	ACCTTCCAGAATATGGAGCCT	AGGAACTTGTGGGAGACAGAG
PHF7	GCAGCCACAGACTACATACC	ATTCTGACACCCTTGGACCT
PHF8	CAAGGAAAGAAGCTCTGCCA	ACTGAAGTGGAATGGGCTG
RBBP8	TCAGCCCTTGAATAAATGCTC	AGAACCAGCACTCTTTATGTC
SLC7A2	GATTCCTAGCTTTCCTCGTG	CCATCCAAATGCTGAATCTGAC
SMC2	ATCAGCAAACATAAACGGGAG	AGTCATTGTATCGCTCTTCAG
SPAST	TACTATTTCTCCTACCCGCTG	CTCCTTCTGTCCTGCTTTCTC
STIM2	GACACTTCCCAGGATAGCAG	GTATAAGCAAACCAGCAGCC
TLK1	AAACTACCACAAACATGCCT	AATCCAAGTCATTGCCTTCAC
WDHD1	GCAGCATTCACAATCTACCA	GAGTTCCACACCATGAATCTG
XIAP	TCGAAGTGAATCTGATGCTG	GATATTTGCACCCTGGATACC
ZBTB16	CTCACATACAGGCGACCAC	CACCCTATAGTGCGTCTCCA
ZNF93	CTACTCTGTGTCCTGTGCT	CAACAATACCAAGGAAGACCA
